# Influence factors analysis of COVID-19 Prevention behavior of chinese Citizens: a path analysis based on the hypothetical model

**DOI:** 10.1186/s12889-022-13514-0

**Published:** 2022-06-01

**Authors:** Yun-shan Li, Rui Wang, Yu-qian Deng, Xiao-rong Jia, Shan-peng Li, Li-ping Zhao, Xin-ying Sun, Fei Qi, Yi-bo Wu

**Affiliations:** 1 School Of Public Health, Shandong University, 250012 Shandong, China; 2 Center for Disease Control and Prevention of Qingdao, 175 Shandong Road, 266033 Shandong, China; 3 Xiangya School of Nursing, Central South University, 410000 Changsha, China; 4 The Second Xiangya Hospital, Central South University, 410000 Changsha, China; 5 School of Public Health, Peking University, 38 Xueyuan Road, Haidian District, 100191 Beijing, China; 6 Key Research Base of Philosophy and Social Sciences in Shaanxi Province, Health Culture Research Center of Shaanxi, 712046 Xi’an, China

**Keywords:** COVID-19, Coronavirus disease 2019, Prevention behavior, The structural equation model

## Abstract

**Background:**

Under the outbreak of Coronavirus disease 2019 (COVID-19), a structural equation model was established to determine the causality of important factors that affect Chinese citizens’ COVID-19 prevention behavior.

**Methods:**

The survey in Qingdao covered several communities in 10 districts and used the method of cluster random sampling. The research instrument used in this study is a self-compiled Chinese version of the questionnaire. Of the 1215 questionnaires, 1188 were included in our analysis. We use the rank sum test, which is a non-parametric test, to test the influence of citizens’basic sociodemographic variables on prevention behavior, and the rank correlation test to analyze the influencing factors of prevention behavior. IBM AMOS 24.0 was used for path analysis, including estimating regression coefficients and evaluating the statistical fits of the structural model, to further explore the causal relationships between variables.

**Results:**

The result showed that the score in the prevention behavior of all citizens is a median of 5 and a quartile spacing of 0.31. The final structural equation model showed that the external support for fighting the epidemic, the demand level of health information, the cognition of (COVID-19) and the negative emotions after the outbreak had direct effects on the COVID-19 prevention behavior, and that negative emotions and information needs served as mediating variables.

**Conclusions:**

The study provided a basis for relevant departments to further adopt epidemic prevention and control strategies.

## Background

Public health emergency events refer to a sudden outbreak of a major infectious disease epidemic, a group illness of unknown cause, or a major food or occupational poisoning that may or have caused a public health hazard to the whole society [1]. The outbreak of Coronavirus disease 2019 (COVID-19) in December 2019 is a major public health emergency, which poses a major challenge to the health system in China. China has controlled the epidemic to a great extent and has entered a stage of normalized epidemic prevention nowadays, because the Chinese government responded to the COVID-19 epidemic in a highly centralized and efficient way, public epidemic prevention also plays an important role in the overall containment of COVID-19 [2, 3].

The effective behavior prevention intervention must be based on the corresponding theoretical basis [4]. There have been many kinds of research on the influencing factors of health protection behavior, and some generally accepted theories and models have been developed to explain such behavior, such as health belief model [5], planned behavior theory [6], information motivation behavior skill model [7], social cognitive theory [8], etc. Although the mechanism behind these theories is different, the influencing factors for integrating them are individual, psychology, and environment. The 3 factors are taken as the basis and combined with the background of COVID-19, We have chosen the external support for fighting the epidemic, the needs level of health information, the cognition of COVID-19 and the negative emotions after the outbreak to construct a structural equation model for explaining COVID-19 prevention behavior [9].

### Analysis Framework and Research Hypothesis

#### External support

Environmental factors are subjective norms, which means that individual behavior will be affected by the surrounding social environment. Environmental factors include external support in to fight against the epidemic. Sufficient external support can significantly improve people’s confidence and adaptability in responding to the epidemic [10]. Many reports have shown that the performance and effectiveness of the Chinese government and institutions in epidemic prevention and control work brings a great sense of security to the public. Social support of the social cognitive theory is widely used and practiced in individual health behavior change [8]. Social support affects disease control through encouraging healthy behavior and modulating effects by reducing the effects of acute and chronic stress on health and helping patients cope with stress resulting from the disease [11]. Therefore, the hypothesis is put forward:

- H1: the external support to fight against the epidemic would have a positive impact on the prevention behavior.
- H2: the external support to fight against the epidemic would have a negative impact on the negative emotions.

#### Negative emotions

Psychology is the brain’s subjective response to objective reality. Psychological factors include negative emotions after the outbreak. Research has shown that emotional states and behavioral efficiency are on a “U” shaped curve, with appropriate levels of emotion promoting behavior and increasing its efficiency, while when emotional states exceed a certain threshold, they can have a hindering effect on behavior [12]. In the face of a severe epidemic situation, the public may have strong negative stress reactions and adopt irrational behavior, while negative emotions prompt the public to search for more health information [13]. Thus, we hypothesized the following.

- H3: negative emotions would have a positive impact on information needs.
- H4: negative emotions would have a negative impact on prevention behavior.

#### Cognitive and information needs

Individual factors include cognitive and information needs. Cognition refers to people’s understanding and view of things. Human behavior is dominated by consciousness, and cognition will inevitably affect their behavior [14], the more comprehensive the cognition of diseases, the less the risk of negative emotions such as anxiety and depression [15]. The impact of cognitive barriers is mainly negative because they not only give rise to negative reactions such as frustration but also block, limit or hamper information seeking [16], highly cognitive people have rich experience in obtaining information, and are more likely to find health information online. Health information is a key determinant of healthy behavior [7], People seeking disease prevention information showed greater likelihood to perform disease prevention behaviors without intentions to perform health promotion behaviors [16], The survey conducted among Zhihu users who have participated in Q&A related to COVID-19 shows the indirect effects of health information seeking on preventive behavior were greater among those with a high level of health information efficacy, which supports indirect effects of information needs [12]. The hypothesis is:

- H5: cognitive situation would have a positive impact on prevention behavior.
- H6: cognitive situation would have a negative impact on negative emotions.
- H7: cognitive situation would have a positive impact on information needs.
- H8: information needs would have a positive impact on prevention behavior.

**Fig. 1:**
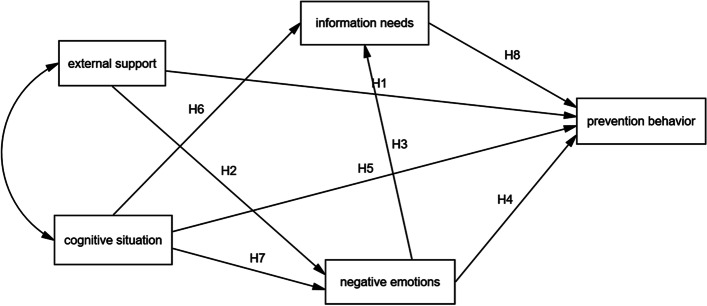
Hypothetical path model

Based on our research hypothesis, we can get the hypothetical path model as shown in Fig. 1.

In this study, we aimed to explore influence factors and the mesomeric effect between different variables of COVID-19 prevention behavior of Chinese citizens. Also, we further identified the causal relationships among the significant factors affecting their prevention behavior by developing a structural equation model, to provide a basis for the public health prevention of COVID-19. Relevant departments could adopt epidemic prevention and control strategies based on results.

## Methods

### Participants and data Collection

The target population of our study is the citizens of Qingdao. It was considered along with 5% precision with a two-sided 5% significance level and 95% power. Besides, the dropout rate was estimated at around 10%. Thus, the minimum sample in this research was 1173. The study used the method of cluster random sampling. Several communities in Shinan District, Shibei District, Huangdao District, Laoshan District, Licang District, Chengyang District, Jimo District, Jiaozhou District, Pingdu City and the Laixi City of Qingdao were randomly selected. We followed strict inclusion and exclusion criteria to select participants. Inclusion criteria for this study included: (1) Participants have lived in Qingdao for a long time; (2) Participate in the study voluntarily and fill in the informed consent form; (3) Participants can complete the online questionnaire by themselves or with the help of the online investigator. Exclusion criteria include: (a) Those who are participating in other similar research projects; (b) People who are unwilling to cooperate.

We recruited investigators to conduct multi-center questionnaires collection, all of whom received standardized and unified training. After introducing themselves and clarifying the research objectives, the investigator distributed research questionnaires among the residents of the community online and the residents filled in the questionnaires by themselves through links Write a questionnaire. Informed consent to take part in the study was obtained from each subject by setting relevant questions before conducting the online survey. After collecting the questionnaire, we carried out the logical inspection of the recovered questionnaire, and eliminated the unqualified questionnaire. We also filtered the IP address to avoid filling in the questionnaire repeatedly and the questionnaire whose time is less than 100 s to ensure the quality of the data. 1218 questionnaires were distributed and 1215 were collected between February 4, 2020, and February 13, 2020. The final analysis included 1188 questionnaires.

### Research instrument

The research instrument used in this study is a self-compiled Chinese version of the questionnaire, which was designed by 17 experts from public health, psychology, sociology, health science popularization and other fields to hold two questionnaire discussion meetings on January 28 and February 2, 2020, respectively. We rigorously evaluated and modified the questionnaire questions, and finally obtained the questionnaire for use. The contents of the questionnaire consist of three parts, which were designed based on the research purpose and the hot issues of public prevention of COVID-19.

#### Demographic and sociological characteristics of the participants

This part of the questionnaire includes their gender, age, nation, district / county-level city, highest education background, marital status, family per capita monthly income, occupation, etc.

#### COVID-19 Prevention behavior

This part associated to the prevention behavior of COVID-19 includes 13 items: (1) Do not contact, purchase and eat wild animals; (2) Refrain from visiting relatives or traveling in the epidemic area; (3) Avoid close contact with people with respiratory disease symptoms; (4) Reduce traveling relatives, friends, and dinner parties; (5) Those who have lived and traveled in the epidemic area within two weeks should be isolated at home by themselves; (6) Prevent going to densely populated public places; (7) Adhere to safe eating habits, such as thoroughly cooking meat and eggs; (8) Maintain indoor cleanliness and open windows frequently for ventilation; (9) Keep hands clean (including washing hands properly); (10) Pay close attention to fever, cough and other symptoms, do a good job in health monitoring; (11) Cover your mouth and nose with cough and sneeze; (12) Take the initiative to wear a mask when suspicious symptoms appear and seek medical attention promptly; and (13) select and wear a mask correctly. A 6-point scale was used for each question to classify protective behaviors according to the diffusion of innovation theory [17] into innovators (early action and persuasion), early adopters (early action and personal evaluation), early public (after action), late public (problematic but action), and laggards (resistance). The scores of each item from “not applicable” (0 points) to “be the first to act and persuade others” (5 points). Each item is scored positively. Finally, the average score of each item is calculated, and the higher the score, the better the participants ' prevention behavior against COVID-19.

#### Influence factors of COVID-19 Prevention behavior

The third part of the questionnaire was designed based on the analysis framework, including four dimensions: external support, cognitive situation, information needs and negative emotions.

##### External support

It is a scale in which participants rate the extent to which the medical and health system, frontline medical personnel, news media, government headquarters, transportation departments, and netizens have played an active role in the fight against the COVID-19 epidemic. The lowest score of 0 means “Feeling the lowest level of support for the epidemic prevention and control of this group”, and the highest score of 10 means “Feeling the highest level of support for the epidemic prevention and control of this group”. Each item is scored positively. The final calculation results in an average of the evaluation scores for each sector. The higher the score, the higher the level of external support.

##### Cognitive Situation

The cognitive situation contains 10 items. (1) Severe acute respiratory syndrome coronavirus 2 (SARS-CoV-2) is sensitive to ultraviolet light and heat; (2) only alcohol can effectively inactivate SARS-CoV-2; (3) the source of infection is mainly symptomatic patients; (4) the sources of infection are all people returning from Wuhan; (5) COVID-19 is mainly transmitted by respiratory droplets. (6) COVID-19 is not transmitted by contact; (7) people of all ages are generally susceptible; (8) the home isolation period for suspected infected persons is 14 days; (9) if infected, there will be symptoms; and (10) the symptoms of COVID-19 are similar to those of influenza. Scores were calculated based on the correctness of the answers to the 10 questions. Each question was scored 1 point for a correct answer and 0 points for an incorrect or unclear answer. Accumulate the total scores of the 10 questions. The higher the score, the higher the respondent’s recognition of COVID-19.

##### Information needs

The information needs consist of nine items. (1) scientific understanding of COVID-19; (2) epidemiological characteristics of COVID-19; (3) methods of disinfection of COVID-19; (4) knowledge of disease prevention; (5) distinction between COVID-19 and other respiratory diseases such as influenza; (6) clinical manifestations after infection; (7) severity of COVID-19; (8) treatment of COVID-19; and (9) vaccine accessibility. Each item was scored positively using a 5-point scale ranging from not at all necessary (1 point) to very necessary (5 points). Finally, the mean score for each item was calculated. Finally, the mean score for each item was calculated. The higher the score, the greater the information needs of the participants.

##### Negative emotions

Considering the special psychology of residents under the epidemic such as anxiety, fear, depression, compulsion, and suspicion [18, 19], negative emotions were referred to the Generalized Anxiety Scale (GAD-7) [20], Patient Health Questionnaire (PHQ-9) [21], and Symptom Self-Rating Scale (SCL-90) [22], containing six items as follows: (1) lack of energy or interest in doing things; (2) feeling depressed, frustrated, or hopeless; (3) repeatedly washing hands and scrubbing things, but always feel that they are not clean enough; (4) feel more nervous than before in places where people gather; (5) often suspect that they or their family members have been infected; and (6) often worry about the impact on their future lives. A 5-point scale was used for each item, ranging from “not at all” (1 point) to “almost every day” (5 points). Each item was scored positively. The final calculation was the average of the evaluation scores for each sector. The higher the score, the higher the level of negative emotions of the participants.

#### Reliability and validity test of the Questionnaire

The content validity of the questionnaire was confirmed using quantitative validity methods. Cronbach’s α was used to measure the internal reliability of the item. External support for fighting the epidemic (α = 0.835, 95%CI 0.820–0.849), demand level of health information (α = 0.959, 95%CI 0.955–0.962), COVID-19 prevention behavior (α = 0.941, 95%CI 0.936–0.946), and cognition of COVID-19 (α = 0.604, 95%CI 0.569–0.638), negative emotions (α = 0.795, 95%CI 0.777–0.813). The reliability of the questionnaire was within the acceptable range. The internal consistency Cronbach’s α coefficient was above 0.60, Kmo value is 0.917 > 0.6. Through Bartlett spherical test, the cumulative variance interpretation rate is 55.221%, which was consistent with our structural framework and had good structural validity. In conclusion, the questionnaire design was reasonable and the evaluation of reliability and validity was good.

### Statistical analysis

IBM SPSS Statistics 22.0 was used for descriptive analysis of the general characteristics of the citizens. The normality of the data of prevention behavior was tested before analysis, and the S-W(Shapiro-Wilk) test results and histogram were both expressed as skewed distribution (*p* < 0.001), so the prevention behavior was expressed by the median ± quartile interval. Residual tests showed insignificant linear trends between the variables and the data did not meet the requirement for chi-squared residuals, making this study unsuitable using linear regression for correlation testing [23, 24]. We use the rank sum test in the nonparametric test (Wilcoxon Mann-Whitney test for two-sample data and Kruskal Wallis test for multi-sample data) to test the influence of citizens’ basic sociodemographic variables on prevention behavior, and use the rank correlation test(Spearman correlation) to analyze the influencing factors of prevention behavior. IBM AMOS 24.0 was used for path analysis with the generalised least squares (GLS) method for parameter estimation [25, 26], including estimating regression coefficients and evaluating the statistical fits of the structural model, to further explore the causal relationships between variables.

## Results

### Sociodemographic characteristics and Rank Sum Test results

The characteristics of the 1188 participants in the study are presented in Table 1. The number of female citizens was more than twice the number of males. Most of the citizens were married (74.16%). Around 63.64% of them never drink wine. The majority of the citizens (90.74%) reported no religion. The scores in the prevention behavior of all citizens varied from 0 to 5, with a median of 5 and a quartile spacing of 0.31.

**Table 1:** Results of Descriptive and Rank Sum Test

Variables	Variable Categories	Total		prevention behavior		H/z	*p*
		n	%	Mean	Median ± interquartile range		
Gender	male	378	31.82	4.593	5.00 ± 0.62	-3.141	0.002
	female	810	68.18	4.772	5.00 ± 0.23		
Age	≤ 25	196	16.50	4.560	5.00 ± 0.46	11.761	0.019
	26–30	113	9.51	4.645	5.00 ± 0.62		
	31–40	393	33.08	4.760	5.00 ± 0.23		
	41–50	391	32.91	4.776	5.00 ± 0.31		
	≥ 50	95	8.00	4.684	5.00 ± 0.38		
Educational background	Junior high school and below	251	21.13	4.626	5.00 ± 0.31	8.264	0.041
	high school	133	11.20	4.766	5.00 ± 0.08		
	Junior College	281	23.65	4.762	5.00 ± 0.31		
	Undergraduate	375	31.57	4.740	5.00 ± 0.38		
	Doctor’s/ Master’s	148	12.46	4.671	5.00 ± 0.60		
Nation	Han nationality	1160	97.64	4.723	5.00 ± 0.31	-0.092	0.927
	Other nationalities	28	2.36	4.401	5.00 ± 0.23		
Marital status	Single	264	22.22	4.638	5.00 ± 0.46	7.002	0.030
	Married	881	74.16	4.740	5.00 ± 0.31		
	Divorced/ Widowed spouse	43	3.62	4.683	5.00 ± 0.08		
Religion	No	1078	90.74	4.720	5.00 ± 0.31	-0.101	0.920
	Yes	110	9.26	4.671	5.00 ± 0.15		
Monthly income per capita	≤ 3000 yuan	255	21.46	4.659	5.00 ± 0.38	0.674	0.714
	3000–4999 yuan	351	29.55	4.704	5.00 ± 0.31		
	≥ 5000 yuan	582	48.99	4.747	5.00 ± 0.31		
Occupation	Personnel of organs / institutions	146	12.29	4.768	5.00 ± 0.23	22.017	0.001
	Medical personnel	90	7.58	4.819	5.00 ± 0.15		
	Professional and technical personnel (excluding medical personnel)	95	8.00	4.672	4.92 ± 0.62		
	Enterprise unit personnel	230	19.36	4.674	5.00 ± 0.38		
	Business and service personnel	180	15.15	4.757	5.00 ± 0.15		
	Student	170	14.31	4.586	5.00 ± 0.54		
	Other employees and nonemployees	277	23.32	4.756	5.00 ± 0.23		
Smoke	Smoke	109	9.18	4.546	5.00 ± 0.62	3.461	0.177
	Quit smoking	68	5.72	4.567	5.00 ± 0.42		
	Never smoke	1011	85.10	4.744	5.00 ± 0.31		
Drinking	Drink	432	36.36	4.687	5.00 ± 0.38	-2.684	0.007
	Never drink	756	63.64	4.732	5.00 ± 0.23		

*p*-value is significant at *p* < 0.05

### Implementation of Prevention actions

More than 75% of the citizens had been able to take early prevention actions and persuade others, including choosing and wearing masks correctly, coughing and sneezing to cover their mouths and noses, keeping the room clean, frequently opening windows and ventilating, avoiding going to densely populated public places, etc., but there are still a few people (about 1.0%) who resist taking prevention actions. Further analysis of the proportion of innovators in various behaviors revealed that more than 83.1% of innovators adopted short-term preventive behaviors such as avoiding densely populated public places, reducing family visits and gatherings, and avoiding visiting relatives or traveling to infected areas. For those who make the right choice and wear masks, cough, sneeze, cover their mouths and noses, health monitoring, and other prevention behavior that can form good habits, the proportion was below 82.7% (Table 2).

**Table 2:** Implementation of prevention actions

	innovators(%)	early adopters(%)	early public(%)	late public(%)	Laggard(%)	Not applicable(%)
Q1	87.5	7.8	0.9	0.3	2.4	1.1
Q2	87.5	8.6	1.1	0.8	1.4	0.6
Q3	84.0	11.7	1.8	0.8	1.1	0.6
Q4	83.9	11.9	1.7	0.5	1.0	1.0
Q5	83.8	8.2	0.9	0.9	0.9	5.3
Q6	83.1	11.5	2.5	0.8	1.3	0.8
Q7	82.7	13.4	2.0	0.8	0.3	0.8
Q8	82.5	13.3	2.4	0.6	0.3	0.9
Q9	82.3	13.0	2.9	0.7	0.4	0.8
Q10	82.3	14.0	2.1	0.5	0.3	0.8
Q11	81.6	13.6	2.1	0.9	0.6	1.2
Q12	81.5	10.5	2.8	1.4	0.5	3.3
Q13	77.4	17.2	3.9	0.5	0.3	0.8

The questions represented by the vertical coordinates Q1 to Q13 are: do not contact, purchase and eat wild animals; avoid visiting relatives or traveling in the epidemic area; avoid close contact with people with respiratory disease symptoms; reduce visiting relatives, friends and dinner parties; those who have lived and traveled in the epidemic area within two weeks should be isolated at home by themselves; avoid public places with the dense population; insist on Safe eating habits: cook meat and eggs thoroughly; keep the room clean and open windows frequently for ventilation; keep hands clean (including washing hands properly); pay close attention to fever, cough and other symptoms, do a good job in health monitoring; cover your mouth and nose with cough and sneeze; take the initiative to wear a mask in case of suspicious symptoms and seek medical treatment in time; select and wear a mask correctly

### Correlation among research variables

The correlation matrix among the research variables is presented in Table 3. Information needs positively correlated with prevention behavior (*r* = 0.312, *p* < 0.001). Cognitive situation showed a positive correlation with prevention behavior (*r * = 0.246, *p* < 0.001), and information needs (*r* = 0.230, *p* < 0.001 ), but negatively correlated with negative emotions (*r* = -0.123, *p* < 0.001). Negative emotions showed negative correlation with prevention behavior (*r* = -0.157, *p* < 0.001), and cognitive situation (*r* = -0.208, *p* < 0.001).

**Table 3:** Correlation matrix of research variables

	Z1		X1		Y1		X2		Y2	
	r	*p*	r	*p*	r	*p*	r	*p*	r	*p*
Z1	1.000									
X1	-0.041	0.161	1.000							
Y1	0.312	< 0.001	0.023	0.422	1.000					
X2	0.246	< 0.001	-0.123	< 0.001	0.230	< 0.001	1.000			
Y2	-0.157	< 0.001	-0.002	0.941	-0.019	0.509	-0.208	< 0.001	1.000	

*X1 *cognitive situation, *X2 *external support, *Z1 *prevention behavior, *Y1 *information needs, *Y2 *negative emotions

### Test of study models

The fit indices of the hypothesis model did not satisfy all fit criteria (hypothesis model: χ2/df = 49.325, NFI = 0.806, IFI = 0.809, TLI=-0.979, CFI = 0.802, RMSEA = 0.202), which means the hypothetical model might be overqualified with unnecessary paths among the variables.

**Fig. 2:**
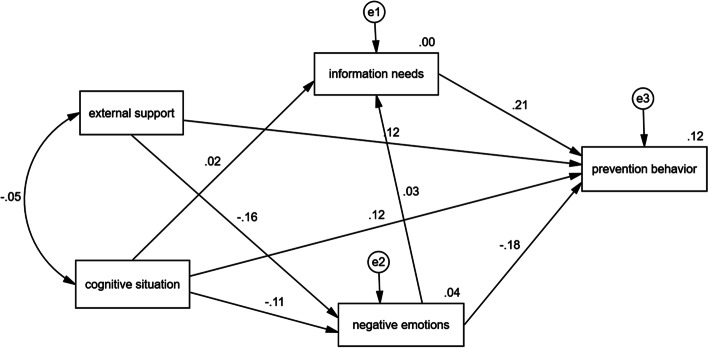
The model before adjustment

According to the path coefficients of the model test results in Table 4, among the 8 hypotheses in the hypothetical model(Fig. 2), 7 (H1, H2, H3, H4, H5, H7 and H8) were confirmed to have statistically significant direct effects. H6, the relationship between the cognitive situation and prevention behavior was not statistically significant and was rejected. Moreover, we find the relationship between the cognitive situation and prevention behavior was not statistically significant external support.

Based on the fit indices of the hypothetical model and test of the hypotheses, it was necessary to revise the model. The paths with no significant statistical effect were eliminated from the hypothetical model. Finally, all of the fit indices of the model were satisfied with the conservative criterion(final model: χ2/df = 1.521, NFI = 0.994, IFI = 0.998, TLI = 0.979, CFI = 0.998, RMSEA = 0.021).

**Fig. 3:**
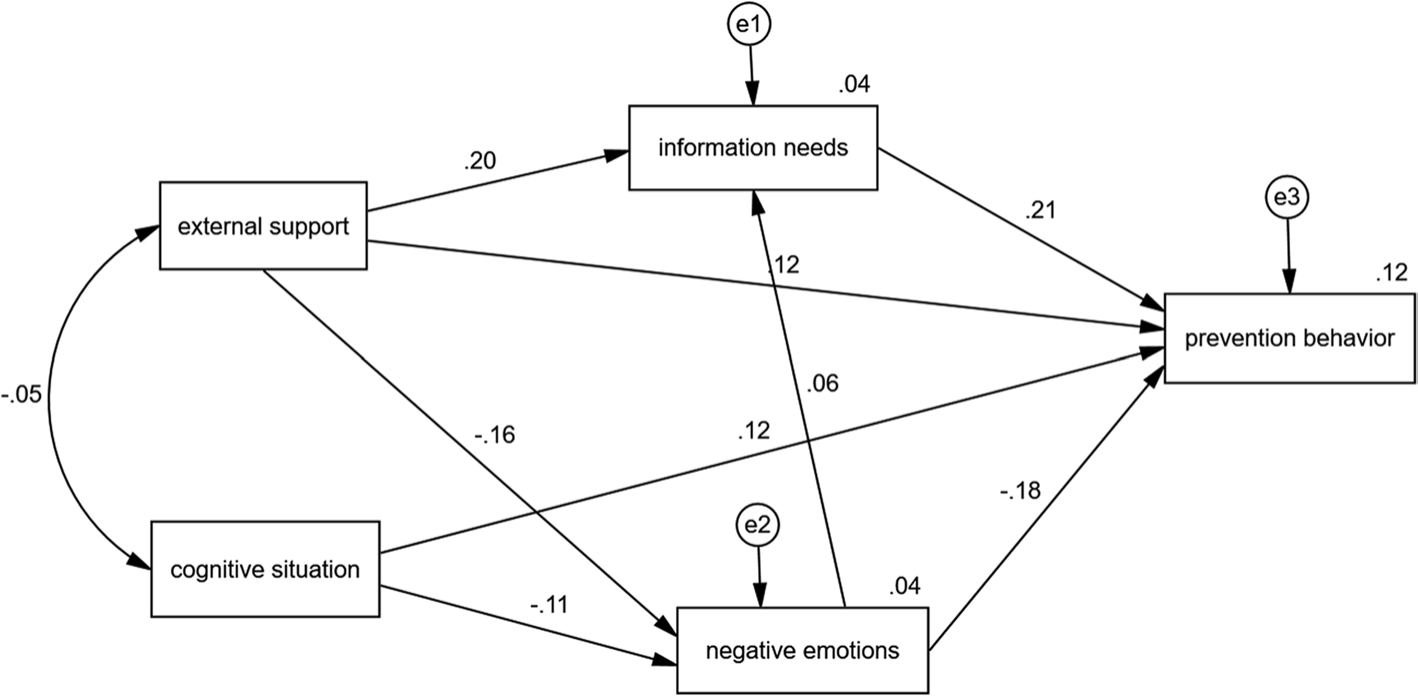
The adjusted model (Standardized Path Estimates)

The final structural equation model of this study showed that external support, information needs, cognitive situation, and negative emotions had direct effects on prevention behavior, while negative emotions and information needs were used as intermediary variables (Fig. 3). The path coefficients of the model test are shown in Table 4.

We found that based on the model shown in Fig. 3, no matter whether any path was deleted or added, the modified model coefficients were not as good as the model. Therefore, we believe that the model shown in Fig. 2 is optimal.

**Table 4:** Path coefficients of the model test

Independent variable		Dependent variable	Nonstandardized Coefficient		Standardized	t	*p*
			β	SE	β		
cognitive situation	→	negative emotions	-0.464	0.119	-0.111	-3.887	< 0.001
external support	→	negative emotions	-0.093	0.017	-0.157	-5.496	< 0.001
external support	→	information needs	0.079	0.011	0.058	7.028	< 0.001
negative emotions	→	information needs	0.038	0.019	0.202	2.012	0.044
information needs	→	prevention behavior	0.224	0.029	0.212	7.639	< 0.001
negative emotions	→	prevention behavior	-0.127	0.019	-0.183	-6.586	< 0.001
external support	→	prevention behavior	0.049	0.012	0.118	4.199	< 0.001
cognitive situation	→	prevention behavior	0.348	0.08	0.12	4.377	< 0.001

## Discussion

Our study indicated that most citizens had been able to take early prevention actions and persuade others, which may be related to the fact that our research sample population is mainly the young and the middle-aged, who are more likely to get and accept more new information [27]. However, the adoption rate of short-term prevention actions was higher than that of long-term ones. It showed that the current epidemic prevention and control propaganda had been in place, and health education should be maintained to make the prevention behavior a living habit of citizens, especially promoting people to vaccinate against COVID-19, as one of the most effective early prevention methods, to increase the vaccination rate [28, 29].

Results of demographic variables indicated that gender, occupation, and drinking were found to be significantly difference in prevention behavior (*p* < 0.01), and age, educational background, and marital status had statistically difference in prevention behavior (*p* < 0.05). The implementation rate of men, aged from 26 to 30 years old, those who have drinking habits and those who are professional technicians (excluding medical personnel) was lower, which might be caused by (1) Women are more likely than men to perceive the risk of disease and take prevention actions; (2) People aged 26 to 30 need to find a job or complete the graduation project, and the epidemic has a greater impact on their lives; (3) People have healthier living habits or a medical background may take the healthy behavior more accurate and timely. Therefore, we should give correct guidance to different groups and behavior.

In our SEM analysis, the external support against the epidemic not only directly had a positive impact on the protection behavior but also took negative emotions and information needs as the intermediary to have a positive impact. external support is crucial to the citizen, helps to promote awareness of the government and the confidence of the medical scientific research institutions in China, have to cope with and overcome the outbreak of the epidemic, because the previous study has found the public measures the government’s handling ability, responsible attitude, communication sincerity, and other factors under the risk situation to form a positive expectation that the government can be relied on, and then take preventive actions [15], Researchers have proved that the external support against the epidemic, especially the support, publicity and guidance from the government, can reduce the risk of negative emotions [9, 30]and increases the degree of information needs [31]. Misinformation on social media will fuel people’s panic regarding the COVID-19 [32, 33]. It is suggested that we should timely convey scientific and positive information and knowledge to the masses looking for the public by using social media [34, 35] and pay attention to the epidemic early warning, and improve the public’s confidence in fighting against the epidemic. At the same time, medical institutions could provide adequate medical protection to the public by vigorously developing new technologies such as telemedicine and health information technology, which are also an important way to provide external support to the public [36, 37].

According to the results of path analysis, the cognitive status had a positive impact on the prevention behavior directly, and it also had a positive impact on the negative emotions as the intermediary. In our study, it was found that there was no significant positive effect of cognitive status on information needs, which may be because people with high cognitive status have learned enough health information and the degree of their needs decreased. Previous studies have indicated that the cognition of diseases is the precondition for an individual to do health behavior [38]. In total, the more comprehensive the public’s cognition of COVID-19, the better the awareness of preventive measures, the better their psychological state, and the more actively they will respond to the changes brought by the epidemic situation and take preventive and control measures, which is consistent with the research of Su’s [39], so after the emergent public health events should be handled in time, strengthen the education of health knowledge and psychological counseling, to improve the cognitive level of the citizens [40].

COVID-19 pandemic was indicated to have a profound and long lasting impact on people’s negative emotions, especially on children and adolescents [41, 42]. The results of SEM analysis indicated that negative emotions not only had a negative impact on the prevention behavior directly but also had a positive impact through the intermediary of information needs. In the face of severe epidemic situations, it is normal to have some stress reactions, but a strong negative stress reaction will lead the public to take irrational actions. For example, the panic buying of Shuanghuanglian Liquid during the epidemic of COVID-19 suggests that psychological counseling should be strengthened during the prevention and control of the epidemic. Public health emergency in the public’s psychological stress reaction stems from a lack of information and misunderstanding [43], but psychological stress reaction can urge citizens to access information, understand the preventive measures,take prevention actions [13], tips on health communication should follow the principle of risk assessment front, avoid causing misunderstanding and public panic.

According to the results of SEM analysis, information needs had a direct positive impact on the protection behavior, and the effect value was the largest. In the outbreak of large-scale epidemic diseases, the public has urgent information needs. Previous studies have shown that meeting the public’s information needs are shown to be associated with better self-efficacy and health-promoting behavior [44]. Therefore, it is necessary to carry out scientific and effective information dissemination, publicity, and education with different groups of people, meet the public’s information needs and guide the public’s cognition and rational prevention and control behavior in time.

Also, we found that there is a difference between the significance of the results of the path analysis and the significance of the rank correlation test. We considered that it may be because the path analysis is a comprehensive analysis involving multiple variables, while the rank correlation only analyzes the linear correlation between two variables. So there may be significant causal paths between certain variables, but their correlation is not significant. So our result is reasonable. However, the results need to be accepted and applied with caution, and further research will be conducted to determine the accuracy of the findings.

The causes and solutions of “*p*-value inflation”, a statistical problem that is often overlooked, are also worth discussing. We consider that there may be a discrepancy between the significance of the statistical results (*p*-value) and the actual results in the rank sum test, rank correlation test and path analysis used in this study, which may be due to several reasons: (1) Limitations of the sampling data. (2) The influence of latent variables. (3) Relatively small differences in the means of different groups. (4) Inadequate construction of the model and limited variables, etc. To try to avoid such errors, the results should be further adjusted during data analysis using appropriate statistical methods to ensure the accuracy of the results. For multiple comparisons of variance either the Bonferroni or Holm method can be chosen [45]. The Bayesian analysis can also be used to provide more accurate parameter estimates for the available data [46].

## Limitations

Our study has several limitations. First, our results only reflected the situations in Qingdao city, and might not represent the whole situation in China. Second, due to the pandemic restriction, collecting data by interviewing participants in-person was not feasible, our survey was an online survey. For those who did not use the Internet or smartphones, some of them were missing, which may cause the sample can’t represent the entire population. Third, Various influencing factors of COVID-19 prevention behavior of the structural model were not comprehensive, the representative was limited. Fourth, our study only investigated the prevention behavior of the subjects against COVID-19 in a limited period and did not conduct a longitudinal comparison of people’s prevention behavior against COVID-19 at different stages of the epidemic. Finally, the correlation coefficients between some of the variables in the results are small, which is a very prominent shortcoming of this study. It indicates that although there are indeed correlations between these variables (significant correlations), the correlations between them is relatively weak. Therefore, we consider that the findings of this study are of limited significance and need to be viewed and applied with caution.

Further research will also be conducted to determine the accuracy of the results in order to improve the reliability of the study findings. We could explore more factors that may influence COVID-19 prevention behavior among general Chinese citizens. In addition, further prospective studies should be conducted to evaluate the relationship between various influencing factors and COVID-19 prevention behavior. Under suitable conditions, we could implement a face-to-face questionnaire survey, which can more effectively prove the research results than online surveys.

## Conclusions

Our findings underscore individual, environmental, and psychological factors interact and influence each other. It is to be noted that most of the effective factors of COVID-19 prevention behavior, the external support for fighting the epidemic, the demand level of health information, the cognition of COVID-19, and the negative emotions after the outbreak can be adjusted and modified by some interventions. We also recommend that the government should release information in a timely and effective manner, the mainstream media should actively provide a communication platform for the government and the public, and report the truth in a timely and accurate manner to meet the information needs of the public, and the relevant departments should actively carry out health education and psychological intervention activities when sudden public health events occur. The implementation of systematic public health strategies, practices and interventions can be used as an effective model for current and future management of public health emergencies, especially for the COVID-19.

## Data Availability

All data generated or analyzed during this study are included in this published article [and its supplementary information files].
